# Allergic reaction to nalbuphine administration in a pre-adolescent patient: a case report and literature review

**DOI:** 10.1186/s12871-026-03864-2

**Published:** 2026-05-02

**Authors:** Shengnan Li

**Affiliations:** 1 Department of Anesthesiology, Union Hospital, Tongji Medical College, Huazhong University of Science and Technology, 1277 Jiefang Avenue, Wuhan, Hubei 430022 China; 2 Institute of Anesthesia and Critical Care Medicine, Union Hospital, Tongji Medical College, Huazhong University of Science and Technology, Wuhan, Hubei 430022 China

**Keywords:** Nalbuphine, Allergic reaction, Pre-adolescent, General anesthesia, Postoperative analgesia, Bronchospasm

## Abstract

**Background:**

Nalbuphine is synthetic agonist-antagonist opioid compound. Allergic reactions to nalbuphine hydrochloride are exceedingly rare. We present a case of hypersensitivity reaction to nalbuphine in a pre-adolescent patient.

**Case presentation:**

A male patient, aged 11 years, underwent general anesthesia for the correction of congenital strephexopodia. Nalbuphine hydrochloride was administered as part of the postoperative analgesia regimen. However, the patient subsequently developed an allergic reaction characterized by pulmonary edema, wheezing, tachycardia and cutaneous rash. Prompt initiation of anti-allergic therapy resulted in significant clinical improvement. The patient was subsequently transferred to the general ward following complete resolution of symptoms.

**Conclusions:**

This case report highlights a hypersensitivity reaction to nalbuphine in pre-adolescent patient, manifesting with both cutaneous and respiratory symptoms. To improve the safety profile of nalbuphine in pre-adolescent populations, further research is necessary to elucidate the underlying mechanisms of such adverse reactions.

## Background

Nalbuphine is a synthetic agonist-antagonist opioid compound, which activates kappa opioid receptors and antagonizes mu opioid receptors [1]. It exhibits moderate analgesia effects and has obvious ceiling effect. Because of mu opioid receptor antagonism and ceiling effect, nalbuphine has very limited risk of inducing receptor-blockade-dependent respiratory failure [2]. Considering its potent analgesia effects and rare adverse effects, it has been recommended for pain management or complications relief derived by other anesthetic drugs for many years among patients older than 18 months [1, 3–6]. Allergic reactions to nalbuphine hydrochloride are rare. We present a case of an allergic reaction to nalbuphine in a pre-adolescent patient with no known allergies. Written informed consent for publication was obtained from the patient’s parent.

## Case presentation

An 11-year-old boy weighing 36 kg was diagnosed with congenital strephexopodia, a rare condition affecting the limbs. His preoperative routine examination was normal, and he had no history of allergies. After being recommended for surgical treatment, he underwent the procedure under general anesthesia. The surgery lasted for two hours, as noted in the anesthesia record. Induction was performed with etomidate (7 mg, iv), sufentanil (8 ug, iv), cisatracurium (7 mg, iv) and ketamine (15 mg, iv), while maintenance was achieved with propofol (10 mg/kg/h) and remifentanil (20 ug/kg/h) by continuous infusion. No intraoperative muscle relaxant was redosed. A 7-mg intravenous dose of nalbuphine hydrochloride was administered for postoperative analgesia ten minutes before surgical closure. The propofol and remifentanil infusions were stopped five minutes prior to surgical closure. Retrospective review of the anesthesia record showed that heart rate increased from 80 bpm to 130 bpm beginning 10 min after nalbuphine and peaking at 15 min. This isolated tachycardia- without hypotension, respiratory signs- was not recognized as anaphylaxis at the time. Despite the return of spontaneous respiration within 30 min after surgery completion, the patient’s consciousness was not regained. Meanwhile, minute ventilation reached 5 L/min. Neuromuscular blockade reversal was not considered. However, during air inhalation, the SpO2 dropped from 100% to 91% within one minute. Simultaneously, a large amount of watery secretion was suctioned from the endotracheal tube. Wheezing was noted on pulmonary auscultation. While wheezing was the dominant auscultatory finding, the possible co-existence of crepitations remained indeterminate. The skin on his face and chest were redder than prior to nalbuphine administration. The heart rate remained around 130 bpm, and his blood pressure stayed stable. Urinary catheter was placed. To suppress excessive respiratory drive and allow controlled ventilation, the patient was sedated with propofol (70 mg loading dose followed by continuous infusion at 10 mg/kg/h), resulting in cessation of spontaneous respiration. Both hydrocortisone(4 mg/kg) and furosemide (5 mg, iv) were administered. Two puffs of salbutamol were administered to relieve bronchospasm. Within minutes, wheezing resolved, and the redness on the chest faded. Heart rate remains unchanged. One and a half hours later, urine output reached 450 ml, and propofol was discontinued. Minute ventilation was similar to the previous level. However, it took 4 min for SpO2 to drop from 100% to 91% during air inhalation this time. The patient was sedated with propofol (70 mg + 10 mg/kg/h), with recurrent respiratory arrest. After another 1.5 h, an additional 350 ml of urine was collected. Propofol was discontinued, and SpO2 remained stable with air inhalation. His consciousness returned, and he was extubated. His heart rate remained elevated above his baseline level, exceeding 110 bpm. His blood pressure remained stable throughout.

## Discussion

Nalbuphine is a synthetic narcotic analgesic, and exhibits analgesia, sedation, and limited respiratory depression. Thus, it has been widely used among pediatric patients for different aims [4, 6–8]. Allergic reactions to nalbuphine have seldomly been reported. Here we present a case of an allergic reaction to nalbuphine in a pre-adolescent patient, underscoring the importance of increased awareness regarding its use in this population. In our case, the allergic reaction primarily involved the cutaneous and respiratory systems, particularly bronchospasm. Cardiovascular manifestations were mainly limited to tachycardia. Notably, hemodynamic stability was maintained throughout the event.

Sedation is one of the most important characteristics of nalbuphine, which has been used to prevent emergence agitation in pediatric patients [4]. Sedation could also be manifested as delayed recovery of consciousness in the post-anesthetic period. In our case, the patient exhibited significantly delayed recovery of consciousness. We analyzed potential factors contributing to delayed recovery of consciousness, including induction protocol, maintenance protocol, intraoperative complications, as well as procedure duration. We could not attribute the delayed awakening to propofol maintenance, as the return of spontaneous breathing occurred 35 min after discontinuation- well within the expected recovery range for propofol in children. Induction protocol and procedure duration could also be excluded. The delayed recovery of consciousness is primarily attributed to nalbuphine administration, which is known to cause sedation in approximately 36% of patients per its prescribing information. The anaphylactic reaction and associated pulmonary edema may have contributed to the clinical picture, but were not the primary cause, as the patient remained unconscious even during mechanical ventilation with SpO2 100%.

In our case, despite elevated respiratory rate and considerable minute ventilation, the patient’s SpO2 could not be maintained with air inhalation. With normal ventilation, the low SpO2 was likely due to diffusion dysfunction or ventilation/perfusion mismatch. He was at supine position and the endotracheal tube was placed at the right position. We are left to attribute his low SpO2 to diffusion dysfunction. Given that watery secretions were suctioned from his trachea, we suspect pulmonary edema despite the absence of X-ray or ultrasound confirmation. Furthermore, improved oxygenation following furosemide treatment supported the diagnosis. Pulmonary edema following nalbuphine administration has been previously reported in adult patients [9, 10]. In addition to pulmonary edema, patient in our case also presented with other clinical symptoms, including wheezing, tachycardia and rash, consistent with a clinical diagnosis of allergic reaction. No blood products or other medications were administered, leading us to conclude that nalbuphine likely caused the allergic reaction. Skin manifestations and airway spasms resolved quickly after hydrocortisone and salbutamol treatment. Since the patient maintained normotension throughout the allergic reaction, with blood pressure consistently comparable to baseline levels, we elected to withhold epinephrine administration. This conservative approach aligns with previous recommendations [11].

Allergic reactions to nalbuphine have been reported in adverse drug reaction on-line information tracking database [12]. However, the characteristics of previously reported cases and mechanism are unknown. Generally, adverse reactions associated with opioids can be classified into two main categories: immune- and non- immune mediated responses [13]. Immune mediated reactions, or hypersensitivity, could be further divided into four different subtypes, with Type I (immediate) and IV (delayed) hypersensitivities being the most common. Non-immune responses include pseudoallergy, idiosyncratic reactions, and intolerance [13]. In pseudoallergy, drugs directly act through the masticate-related G protein coupled receptor X2 [14], activating mast cells, promoting histamine release and causing reactions similar to type I hypersensitivity [15–17]. Both allergic and pseudoallergic reactions are related to histamine release. Previous studies have shown that opioid-induced histamine release rarely involves airways, particularly bronchospasm [18, 19]. Furthermore, evidence suggests that histamine released by opioids administered at normal doses in healthy patients seldom triggers reactions or causes true IgE antibody-mediated anaphylactic reactions [19, 20]. Hypersensitivity to opioids is more likely to occur during anesthesia than when opioids are administered for analgesia [21, 22]. If severe bronchospasm occurs, the reaction is likely to be immune-mediated [19, 23, 24]. In fact, different opioids show varying potential for histamine release as well as distinct anatomical sites where release occurs [25]. Previous study showed that nalbuphine has the potential to promote histamine release in plasma [26]. To date, the characteristics of histamine release induced by nalbuphine have not been clearly defined. In our case, since the manifestations involved both the skin and lungs, particularly wheezing, we can hypothesize that nalbuphine promotes histamine release in both skin and lungs. The adverse effect following nalbuphine administration in our case may be largely attributed to immune-mediated mechanism because of bronchospasm.

Limitations are as follows. First, we did not perform skin prick testing, detect histamine in plasma or tryptase levels to confirm the diagnosis because of safety as well as economic cost. However, clinical manifestations and the effectiveness of anti-anaphylactic treatment strongly support our diagnosis. Second, we did not aggressively pursuing crepitation assessment post-wheezing due to auscultation’s limited accuracy in edema diagnosis [27], the intubated patient’s supine position requiring movement, and adequate existing diagnostic evidence. Third, we recognize the lack of arterial blood gas as a limitation of this report. However, the decision to omit arterial blood gas analysis in hemodynamically stable children was justified by three factors: (a) the patient remained hemodynamic stable with no arterial line in place-obtaining arterial blood gas would have required a separate arterial puncture in a child, (b) institutional protocols minimizing invasive procedures in pediatric cases with clear diagnostic evidence, and (c) alignment with ethical guidelines to reduce avoidable invasive interventions in pediatric patients. In future similar cases, if an arterial line is already present or if the diagnosis is uncertain, arterial blood gas should be obtained. Finally, this study did not quantitatively assess neuromuscular blockade recovery, but the patient’s minute ventilation and clinical signs suggested complete recovery. This is the first reported case of an allergic reaction to nalbuphine in a pre-adolescent. Specially, the patient had no history of allergies. Further studies are needed to explore the underlying mechanism, and to avoid the use of nalbuphine in high-risk patients.

In conclusion, we report a case of nalbuphine-induced allergic reaction in a pre-adolescent. To improve the safety of nalbuphine use in pre-adolescents, further studies are needed to elucidate the potential mechanisms of allergic reactions to nalbuphine.

## Data Availability

On reasonable request, data regarding this patient can be obtained from the corresponding author.
